# *CmFUL1* was potentially involved in fruit elongation in melon

**DOI:** 10.1093/hr/uhaf138

**Published:** 2025-05-21

**Authors:** Lingli Tang, Yuhua He, Bingxue Liu, Mingqian Liu, Yongyang Xu, Jian Zhang, Weihu Kong, Lulu An, Keyun Hu, Jordi Garcia-Mas, Bin Liu, Guangwei Zhao

**Affiliations:** National Key Laboratory for Germplasm Innovation & Utilization of Horticultural Crops, Zhengzhou Fruit Research Institute, Chinese Academy of Agricultural Sciences, Gangwan Road No.28, Guancheng Region, Zhengzhou 450009, China; Zhongyuan Research Center, Chinese Academy of Agricultural Sciences, Hongqiqu Road NO.28, Pingyuan shifan Region, Xinxiang, Henan 453500, China; National Key Laboratory for Germplasm Innovation & Utilization of Horticultural Crops, Zhengzhou Fruit Research Institute, Chinese Academy of Agricultural Sciences, Gangwan Road No.28, Guancheng Region, Zhengzhou 450009, China; National Key Laboratory for Germplasm Innovation & Utilization of Horticultural Crops, Zhengzhou Fruit Research Institute, Chinese Academy of Agricultural Sciences, Gangwan Road No.28, Guancheng Region, Zhengzhou 450009, China; Nanjing Agricultural University, Hankou Road NO.22, Gulou Region, Nanjing, Jiangsu 210000, China; National Key Laboratory for Germplasm Innovation & Utilization of Horticultural Crops, Zhengzhou Fruit Research Institute, Chinese Academy of Agricultural Sciences, Gangwan Road No.28, Guancheng Region, Zhengzhou 450009, China; National Key Laboratory for Germplasm Innovation & Utilization of Horticultural Crops, Zhengzhou Fruit Research Institute, Chinese Academy of Agricultural Sciences, Gangwan Road No.28, Guancheng Region, Zhengzhou 450009, China; National Key Laboratory for Germplasm Innovation & Utilization of Horticultural Crops, Zhengzhou Fruit Research Institute, Chinese Academy of Agricultural Sciences, Gangwan Road No.28, Guancheng Region, Zhengzhou 450009, China; National Key Laboratory for Germplasm Innovation & Utilization of Horticultural Crops, Zhengzhou Fruit Research Institute, Chinese Academy of Agricultural Sciences, Gangwan Road No.28, Guancheng Region, Zhengzhou 450009, China; National Key Laboratory for Germplasm Innovation & Utilization of Horticultural Crops, Zhengzhou Fruit Research Institute, Chinese Academy of Agricultural Sciences, Gangwan Road No.28, Guancheng Region, Zhengzhou 450009, China; Centre for Research in Agricultural Genomics (CRAG) CSIC-IRTA-UAB-UB, Edifici CRAG, Campus UAB, Bellaterra, 08193 Barcelona, Spain; Institut de Recerca i Tecnologia Agroalimentàries (IRTA), Edifici CRAG, Campus UAB, Bellaterra, 08193 Barcelona, Spain; Hami-Melon Research Center, Xinjiang Academy of Agricultural Sciences, Nanchang Road NO.403, Shayibake Region, Urumqi 830000, China; National Key Laboratory for Germplasm Innovation & Utilization of Horticultural Crops, Zhengzhou Fruit Research Institute, Chinese Academy of Agricultural Sciences, Gangwan Road No.28, Guancheng Region, Zhengzhou 450009, China; Zhongyuan Research Center, Chinese Academy of Agricultural Sciences, Hongqiqu Road NO.28, Pingyuan shifan Region, Xinxiang, Henan 453500, China

## Abstract

Melon (*Cucumis melo* L.) is a fruit crop in the world; fruit size and fruit shape are major traits for melon quality. Fruit length is a crucial indicator affecting fruit size and shape, but few genes regulating this trait have been identified. Here, we identified the transcription factor CmFUL1 (FRUITFULL) as a candidate for regulating fruit length using genome-wide association analysis (GWAS) and phylogenetic analysis. *CmFUL1* is mainly expressed during flower and ovary development by tissue-specific expression. Transcriptional analysis revealed that *CmFUL1* expression levels exhibited a negative correlation with fruit length across diverse melon germplasm. Furthermore, functional characterization demonstrated that CmFUL1 acts as a negative regulator of fruit elongation, *CR-Cmful1* mutants generated by CRISPR-Cas9 showing enhanced longitudinal fruits. This repressive role was evolutionarily conserved, as heterologous overexpression of *CmFUL1* in tomato consistently inhibited fruit elongation. Collectively, the results suggested that *CmFUL1* is a candidate gene involved in regulating fruit length in melon, and provided genetic resources for molecular breeding of melon.

## Introduction

In the Cucurbitaceae family, melon is a significant economic crop ranking as the ninth most popular fruit globally. Melon was classified into two subspecies, *Cucumis melo* ssp. *agrestis* (hereinafter referred to as *agrestis*) and *C. melo* ssp. *melo* (hereinafter referred to as *melo*) based on ovary pubescence, and further divided into 11 varietas or 19 groups [1, 2]. Melon is diverse with individual fruit weights ranging from 0.01 to 10 kg (a nearly 1000-fold difference) and lengths varying from 5 to 200 cm (a nearly 40-fold difference) compared to other species in the Cucurbitaceae family, making them an excellent crop for research in fruit development [3–5]. Fruit size is determined by both cell quantity and volume, which are primarily regulated by cell length. The development process of melon includes the growth period of fruit cell division, fruit expansion, and fruit ripening [6, 7].

Fruit size, comprising length, width, and shape, is a complex quantitative trait influenced by multiple genes [8–11]. A meta-analysis of fruit-related quantitative trait loci (QTLs) in cucurbits identified 26 fruit size QTLs, 33 fruit shape QTLs, and 19 fruit weight QTLs that colocalized across multiple melon populations [12]. Studies found the significance of the *FRUITFULL* (*FUL*), *IQD/SUN*, *CYP78A*, *CNR*, and *OVATE* gene family in fruit development [13–15]. A comparative genomic approach was used to identify homolog genes of *SUN* and *OVATE* in melons [16].

Despite extensive identification of QTLs associated with melon fruit morphology, only a limited number of genes governing these traits have been functionally characterized to date [9]. Fruit development in melon is coordinately regulated by phytohormones including gibberellins and brassinosteroids, as well as various metabolic pathways. Notably, the 3-hydroxy-3-methylglutaryl coenzyme A reductase (CmHMGR) plays a pivotal role in this process by catalyzing the rate-limiting step in mevalonic acid biosynthesis, a crucial precursor for isoprenoid metabolism [17]. The *CmHMGR* enzyme is involved in regulating melon size, specifically through influencing cell proliferation within melon pericarp [18]. The *CmACS-7* gene, encoding a 1-aminocyclopropane-1-carboxylate oxidase (ACS), has been demonstrated to play multiple roles in regulating andromonoecy, and affecting fruit length and shapein melon [19–21]. AtFRUITFUL (AtFUL), a transcriptional repressor in the MADS-box family that regulates silique elongation in *Arabidopsis*, has functional homologs in tomato. Specifically, SlFUL1 and SlFUL2, the tomato orthologs of AtFUL, interact with the RIPENING INHIBITOR (RIN) transcription factor to coordinately regulate fruit ripening processes during development [22–24]. Furthermore, *ACC SYNTHASE2* (*ACS2*) was decreased in the *Slful1/Slful2* mutant, which suggested that SlFUL proteins affect tomato ripening by fine-tuning ethylene pathway [25].

Genome-wide association analysis (GWAS) and QTL mapping are effective methods for elucidating the gene controlling of fruit traits in plants [26, 27]. Previous integrative studies employing GWAS, QTL mapping, and transcriptome profiling have successfully identified *CmOFP13* as a key regulatory gene associated with melon fruit shape determination. This multi-omics approach has not only revealed the genetic architecture underlying fruit morphology but also established a molecular framework for investigating the regulatory mechanisms controlling longitudinal fruit development in cucurbit species. [28, 29]. Using these strategies, researchers have identified the genetic loci that control traits such as yield, monoecy, color, fruit firmness, soluble sugar content, acidity, and aroma in melon fruits ([5, 30–33]; Santo [34, 35]).

In this study, we conducted a GWAS using a diverse panel of melon accessions to systematically identify single nucleotide polymorphisms (SNPs) associated with fruit length variation. This study pursued two overarching objectives: [36] to pinpoint QTLs and characterize putative candidate genes underlying phenotypic variation in fruit length, and [22] to elucidate the functional roles of prioritized candidate genes through genetic validation. Our findings not only enhance our understanding of the genetic architecture underlying melon fruit elongation but also provide valuable resources for marker-assisted selection, thereby accelerating the development of melon cultivars with optimized fruit morphology. The genomic resources and functional insights presented in this study lay a foundation for dissecting the molecular mechanisms regulating fruit development in Cucurbitaceae species.

**Figure 1:**
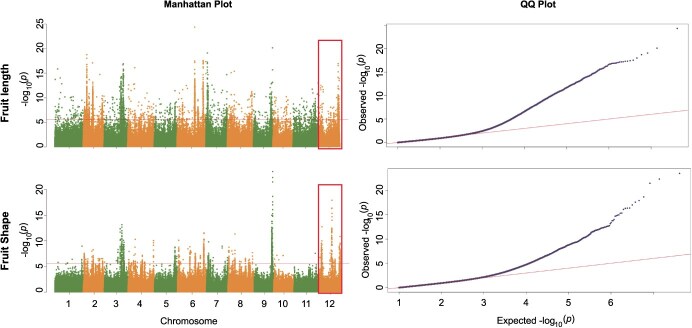
Independent selection in fruit traits among the 1175 melon accessions. Ordinate axis indicates fruit traits. And abscissa axis indicates the chromosome 12. Horizontal lines indicate the genome-wide threshold of selection signals. *P*-value observations for these loci should be consistent with the Manhattan Plot values.

**Figure 2:**
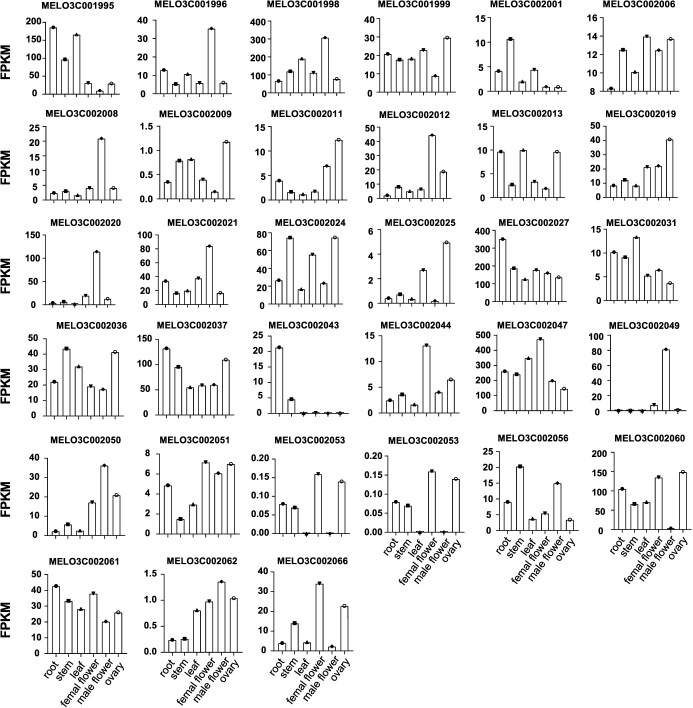
The expression pattern of candidate genes. The candidate gene on the region on the chromosome 12 from the Melon reference genome version 3.5.1.

## Results

### Identification of *MELO3C002050* for regulating melon length

To investigate the genetic basis of fruit length regulation in melon, we conducted GWAS on two correlated traits: fruit length and fruit shape. Utilizing resequencing data and phenotypic records from 1175 melon accessions, our analysis revealed a significant overlapping genomic region on chromosome 12 (25.38–25.84 Mb; reference genome v3.5.1) that showed strong associations with all three traits (Fig. 1, Supplementary Table S2). Notably, this critical interval coincides with previously reported QTLs FS6 and FS12.1, which have been associated with single fruit weight and shape parameters in independent studies [36, 44, 45]. The convergence of GWAS signals from multiple fruit-related traits in this genomic region strongly suggests its pivotal role in regulating melon fruit morphology.

There were 35 genes on this region on chromosome 12 according to the melon v3.5.1 genome reference, the detailed descriptions of these candidate genes are in Supplementary Table S3. The transcript level of all genes from the project PRJNA803327 were listed in Fig. 2 [46]. We found that most of these genes were expressed in root, stem, leaf, female flower, male flower, and ovary, while *MELO3C002043* was expressed in root and leaf, and *MELO3C002049* only expressed in flower. Our analysis revealed that *MELO3C002050*, encoding a MADS-box transcription repressor expressed in melon ovaries, represents the ortholog of CsFUL1 in *C. melo*. Notably, MADS-box transcription factors have been extensively documented as crucial regulators in plant developmental processes and fruit maturation pathways. This functional conservation, coupled with our expression profile data, strongly supports our hypothesis that *MELO3C002050* may serve as a key regulatory gene mediating fruit elongation in melon.

### Phylogenetics and expression pattern of *MELO3C002050*

**Figure 3:**
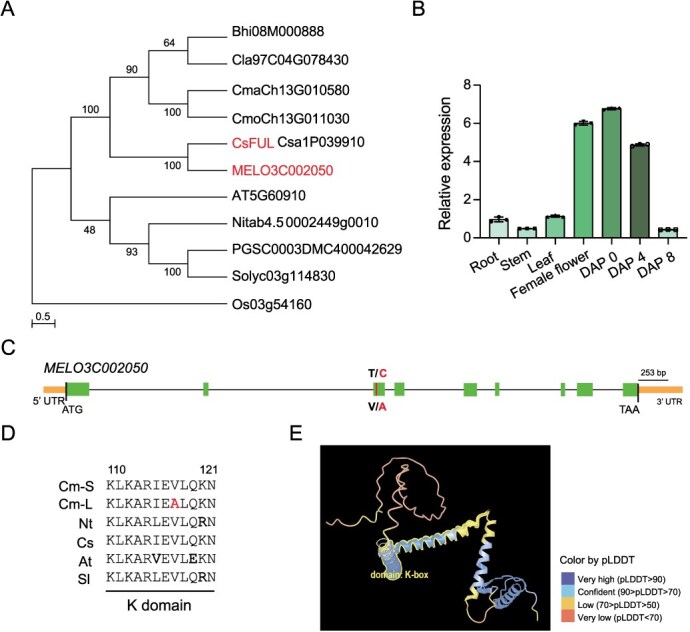
Evolution and expression pattern of *CmFUL1.* (A) The phylogenetic tree of *CmFUL1* and homologous genes in cucumber (Csa1p039910), *Arabidopsis* (AT5G60910), tobacco (Nitab4.50002449 g0010), tomato (Solyc03g114830), rice (Os03g54160), potato (PGSC0003DMC400042629), wax gourd (Bhi08M000888), watermelon (Cla97C04G078430), and +pumpkin (CmoCh13G01030, CmaCh13G010580). (B) The relative expression of CmFUL1 in tissues by RT-qPCR. CmACTIN1 was used as an internal control, and three biological replicates were used. (C) The gene structure of *CmFUL1*. (D) The K-domain of CmFUL1 orthologs in short melon (S), long melon (Long), tobacco (Nt), cucumber (Cs), *Arabidopsis* (At), and tomato (*Solanum lycopersicum* L.). (E) The protein structure of CmFUL1.

To further clarify the phylogeny of *MELO3C002050*, homologous genes of *MELO3C002050* in cucumber, *Arabidopsis*, tobacco, tomato, and other crops in the NCBI database were used to build a phylogenetic tree with MEGA 7.0; *MELO3C002050* has a closer relationship with the homologous gene in cucumber (Fig. 3A). The protein sequence identity of melon *MELO3C002050* and cucumber *Csa1P039910* (*CsFUL1*) was 92.47%, so we named *MELO3C002050* as *CmFUL1* (hereafter *CmFUL1*) (Fig. 2A). Intriguingly, *CsFUL1* has been confirmed to regulate fruit length in cucumber [37]. The tissue-specific expression pattern of CmFUL1 showed that it had a high expression in flower, the day of pollination (DAP 0), and the early fruit-setting days through qRT-PCR (Fig. 3B).

Furthermore, genomic analysis revealed that *CmFUL1* comprises nine exons, with a T-to-C SNP identified in the third exon that results in a non-synonymous valine-to-alanine substitution (Val117Ala) (Fig. 3C). Comparative genomic analysis revealed that a single amino acid substitution was uniquely conserved in the long germplasm of melon, in contrast to the homologous sequences observed in tobacco, cucumber, tomato, and *Arabidopsis* (Fig. 3D). Notably, a single amino acid substitution within the K-domain of CmFUL1 was predicted through protein structural analysis (MutationTaster: https://www.mutationtaster.org/) to compromise protein stability and protein folding (Fig. 3E). While this mutation demonstrates clear structural implications, the functional significance of other identified variants remains to be elucidated. This conserved amino acid alteration in the predicted functional domain prompted us to hypothesize that *CmFUL1* may play a regulatory role in melon fruit morphological development, potentially through protein structural modification.

**Figure 4:**
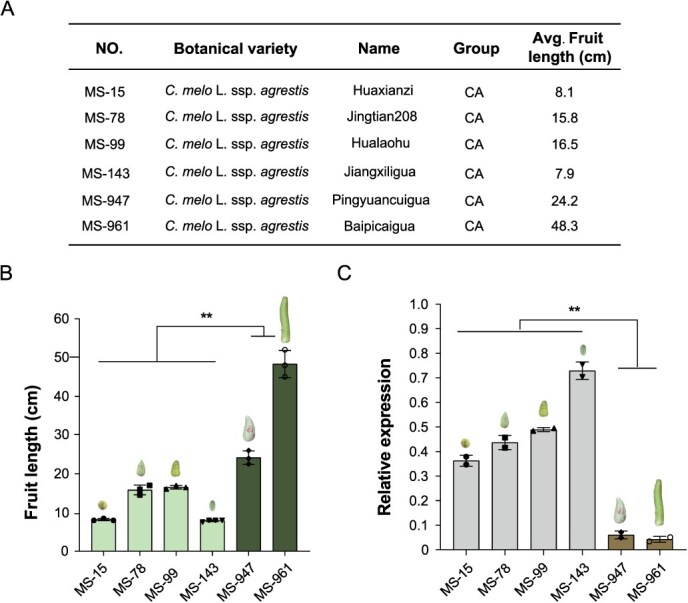
Correlation between fruit length and *CmFUL1* expression. (A) The information of six accessions in the subspecies *agrestis.* (B) The fruit length of six accessions in the subspecies *agrestis*. (C) The expression level of *CmFUL1* in the six species by qRT-PCR. *CmACTIN1* was used as an internal control, and three biological replicates were used, error bars indicate the standard error of the mean. ^**^P < 0.01.

Furthermore, we explored the expression of *CmFUL1* 15 days after pollination (DAP) fruits of six melon accessions with contrasting fruit lengths in the subspecies *agresis*, including (MS-15 (8.1 cm), MS-78 (15.8 cm), MS-99 (16.5 cm), MS-143 (7.9 cm), MS-947 (24.2 cm), and MS-961 (48.3 cm)), named Huaxianzi, Jintian208, Hualaohu, Jiangxiligua, Pingyuancuigua, and Baipicaigua, respectively (Fig. 4A), and found that there was an opposite correlation between the expression level of *CmFUL1* and fruit length (Fig. 4B and C) (Supplementary Tables S4 and S5). Taken together, these results suggest that *CmFUL1* negatively regulates fruit elongation of melon.

### *CmFUL1* negatively regulates fruit elongation

**Figure 5:**
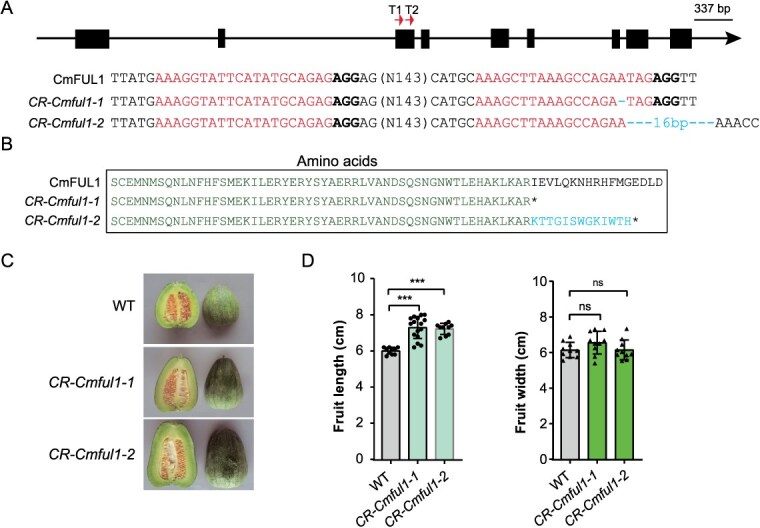
The targets design, phenotype statistics of WT, and *CR-Cmful1.* (A) The gene structure and 16-bp deletion in the fourth exon of WT and *CR-Cmful1*, respectively. (B) Amino acids of *CR-Cmful1* compared to WT. (C) The fruit phenotype of WT and *CR-Cmful1*. (D) The fruit length and width statistics of WT and *CR-Cmful1*. ^***^ means *P* < 0.001, ns means no significant difference.

To further elucidate the functional role of *CmFUL1*, we generated transgenic lines containing one base deletion and 16-bp deletion mutant lines in the coding sequences through CRISPR-Cas9. Successful editing events were verified through PCR amplification and Sanger sequencing analyses (Fig. 5A). The amino acid alterations resulted from a base and 16-base deletion event that induced frameshift mutations within open reading frames, subsequently leading to premature translation termination (Fig. 5B). Comparative phenotypic analysis demonstrated statistically significant increases in fruit length (*P* < 0.001) in *CR-Cmful1* mutant lines relative to wild-type (WT) controls. Notably, no significant variation was observed in fruit width between mutants and WT plants. (Fig. 5C and D; Supplementary Table S6). These findings demonstrate that *CmFUL1* acts as a negative regulator of fruit elongation in melon.

**Figure 6:**
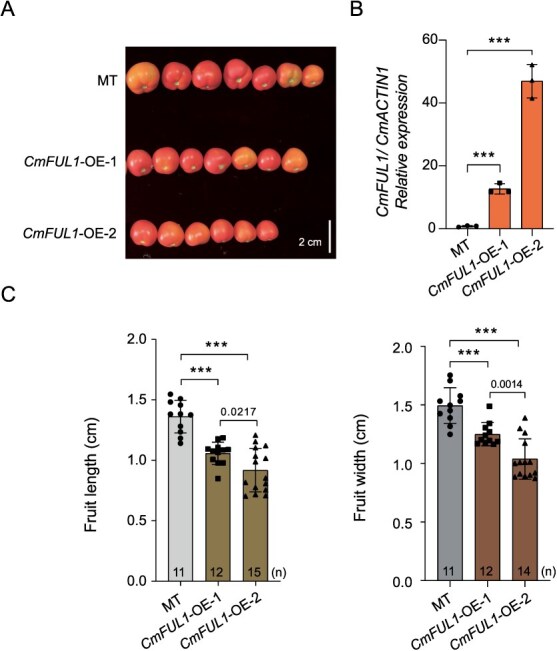
Functional validation of *CmFUL1.* (A) The fruit phenotype of *CmFUL1* overexpression lines (*CmFUL1-OE-1* and *CmFUL1-OE-2*). (B) qRT-PCR expression levels of *CmFUL1* in *CmFUL1-OE-1* and *CmFUL1-OE-2* lines and MT. *SlACTIN1* was used as an internal control, and three biological replicates were used. Error bars indicate the standard error of the mean. ^**^P < 0.01, ^***^P < 0.001. (C) The fruit length and fruit width of *CmFUL1-OE* lines and MT. The number of replicates was 11, 12, 15, and 11, 12, 14, respectively. Error bars indicate the standard error of the mean. ^***^P < 0.001.

To identify the function of *CmFUL1*, we overexpressed it in the tomato accession ‘MicroTom’ (MT), and observed that the fruits of the overexpressed lines *CmFUL1* (*CmFUL1-OE-1* and *CmFUL1-OE-2*) were significantly smaller than MT (Fig. 6A). The transcription levels of *CmFUL1* in *CmFUL1-OE* lines were significantly higher than that in MT (Fig. 6B). Notably, transgenic tomato lines overexpressing *CmFUL1* exhibited significant reductions in both longitudinal (fruit length) and lateral (fruit width) dimensions compared to WT MT plants (Fig. 6C; Supplementary Tables S7 and S8). These results suggest that *CmFUL1* negatively regulates fruit elongation when it was overexpressed in tomato.

## Discussion

We identified a gene *CmFUL1* regulating fruit length using GWAS and phylogenetic analysis on chromosome 12. An SNP mutation (T–C) was found in the third exon of *CmFUL1* and tissue-specific analysis revealed that *CmFUL1* is mainly expressed in flower and ovary development. While *CmFUL1* was the homolog of *CsFUL1*, which belonged to MADS-box family and was determined as a negative regulator of cucumber length. *CsFUL1* mainly regulated the transcript level of *PIN-FORMERD1* (*PIN1*) and influenced the auxin accumulation in cucumber [47]. In this work, we also found a negative correlation between the transcript level of *CmFUL1* and melon length in six contrasting melon accessions. Furthermore, we found that the length of melon was elongated in the *CmFUL1* mutants created by CRISPR–Cas9 technology, and fruit smaller in the *CmFUL1* overexpressed lines in tomato. Additionally, field trials of *FUL* mutant strains in pea have shown that they can be used to improve crop yields [48]. The function of FUL protein regulating fruit length is conserved in vegetable plants with different inflorescence structures.

The *FUL1* and *FUL2* might be produced in an early whole-genome multiplication event, the evolution of the *euFUL* genes were identified probably correlating with the origin of fleshy fruit [49]. There is one FUL in *Arabidopsis*, and two members in tomato, cucumber, and melon. The increased number of FUL homologous identified in plants meaning functional redundancy and divergence among these genes. Single- and double-mutant alleles of *FUL1* and *FUL2* created through CRISPR-Cas9 show that the two proteins have redundant functions in fruit ripening and reveal an extra role for only FUL2 in early fruit development [50]. And we found *CmFUL1* repressed the length of melon fruit also through the single mutant, but the downstream genes and the signaling pathways regulated by CmFUL1 in melon need to be further explored.

Recent studies have shown that *FUL1* is a downstream target gene of STM3 and J2, and these two transcript factors antagonistically regulate inflorescence branching in tomato [51]. Using CRISPR-Cas9-mediated genome editing system, fruit size, color, and ripening were changed in the *RIN*, *FUL1*, and *FUL2* single and multiple mutants, which reflected the redundant and various effects of these genes on fruit development [52]. By integrating protein sequence with function analysis, a key amino acid motif has identified that affects the interaction between the MADS-domain proteins AGAMOUS and SEPALLATA, thereby linking it to multiple biological progresses [53]. Although the CR-CmFUL1 mutants did not exhibit accelerated fruit ripening compared to the wild type (WT) in melon, this finding suggests that CmFUL1 may not play a direct regulatory role in melon ripening progression, unlike its ortholog in tomato which significantly influences the ripening process. More in-depth research is needed to reveal the molecular mechanism of *CmFUL1*.

In this study, we found that the gene *CmFUL1* is associated with fruit length through GWAS. *CmFUL1* is mainly expressed during flower and ovary development by tissue-specific expression. The expression patterns of *CmFUL1* were oppositely related to fruit length in various melon accessions, while *CmFUL1* negatively regulates fruit elongation when we created the *CR-Cmful1* mutants by CRISPR-Cas9 in melon and overexpressed in tomato. Collectively, those results suggested that *CmFUL1* is a candidate gene that played a role in regulating fruit length in melon. The molecular mechanism by which *CmFUL1* regulates melon fruit length deserves additional study.

## Materials and methods

### Plant material

Accessions of melon were sourced from the Zhengzhou Fruit Research Institute (Zhengzhou, China), Chinese Academy of Agricultural Sciences [37]. The accessions were phenotyped for fruit length, fruit shape, and ovary shape according to the Chinese technical specification evaluation for melon [38].

### Genome wide association studies, SNP annotation, and analyses

Sequencing libraries were constructed by genomic DNA, and subsequently sequenced on Illumina HiSeq3000 or the HiSeq2500 [37, 39]. The total number of SNPs was analyzed using the expedited (EMMAX) program, which employs an efficient mixed-model association analysis. Hidden relatedness and population stratification were accounted for using a kinship (K) matrix. The significance thresholds for *P*-values were set at ~2.51 × 10^−6^. Details on the putative QTL intervals derived from the QTL-seq analysis is presented. SNPs located within these QTL intervals were extracted using Bedtools [40]. The Variant Effect Predictor (VEP) tool was employed to annotate the QTL-associated SNPs.

### Sequence alignment and variation calling analysis

All reads were mapped to melon reference genome (Version 3.5.1) for SNP calling [41]. We used the GLFmulti52 to invoke individual SNPs, utilizing the maximum likelihood assess of locus frequency to integrate SNPs across the dataset [42]. The core SNPs were obtained based on allele frequencies and the quality score provided by GLFmulti52. We further filtered through segregation testing to obtain final SNPs that can distinguish any segregation patterns from random sequencing errors based on the sequencing depth of the two putative alleles in different individuals. Permutations were engaged to calculate the significance of allele depth, leaving only loci with *P* < 0.01.

### Plasmid constructs and melon transformation

To generate the *35S:CmFUL1-GFP* transgenic construct, the coding sequence of *CmFUL1* was inserted into the pRI101-GFP vector using an In-Fusion HD Cloning Kit (TransGen Biotech, China) following the manufacturer’s protocol. The recombinant construct was subsequently introduced into the Microtom tomato cultivar through *Agrobacterium tumefaciens*-mediated transformation, followed by standard tissue culture regeneration procedures. The entire genetic transformation process was professionally executed by BioRun Biotechnologies Co., Ltd. (Wuhan, China) using optimized protocols for Solanaceae species transformation.

### CRISPR–Cas9 genome editing and melon transformation

Targeted mutagenesis in melon was performed using the PBS-TPC-Cas9 system developed by Bin Liu. The binary vector was constructed through sequential Golden Gate cloning and In-Fusion cloning following established protocols. Subsequently, the recombinant vector was introduced into melon via *A. tumefaciens*-mediated transformation using standard methods. Regenerated transgenic plants were acclimatized in controlled greenhouse conditions and subjected to molecular characterization. Mutations were confirmed through polymerase chain reaction (PCR) amplification followed by Sanger sequencing, with genotyping primers detailed in Supplementary Table S1.

### Plant phenotyping and imaging

Phenotypic characterization was conducted with T_1_ plants derived from self-pollination. CRISPR-edited melon mutants were first subjected to Basta selection (400 mg l^−1^) followed by PCR genotyping to confirm transgene-free status. Manual measurements and photographic documentation of fruit morphology were systematically performed to record phenotypic variations.

### RNA extraction, gene expression, and real-time PCR analysis

Total RNA was isolated from melon fruit using the RNA Extract Kit (Bioman, Beijing, China) according to the manufacturer’s protocol. The expression level of *CmFUL1* was quantified by quantitative reverse transcription PCR (qRT-PCR). Gene-specific primers for *CmFUL1* were designed using the Primer-BLAST tool on NCBI, and the *CmACTIN* was selected as a stably expressed internal control. All primers (sequences provided in Supplementary Table S1) were synthesized by Sangon Biotech (Zhengzhou, China). RT-PCR was carried out with an All-in-one mix kit (Bioman, Shanghai, China). qRT-PCR detections for the target genes were performed with the SYBR Green FAST Mixture qPCR kit (GenStar, Shenzhen City, China) through the LightCycler480 real-time PCR detection System (Roche Diagnostics, Indianapolis, IN, USA), following by the PCR program of 95°C for 30 s, 40 cycles of 95°C for 15 s and 60°C for 15 s, and finally 72°C for 30 s. Relative gene expression was calculated through the 2^−∆∆CT^ method.

### Transcriptomic resources

The RNA-seq data were from the PRJNA803327 project, focusing on the ‘Hetao’ melon with various tissues, which was used to investigate the transcript levels of *CmFUL1*. The normalized FPKM of the distinct samples were then analyzed using GraphPad Prism 8.

### Statistical analysis

GraphPad Prism8 software were used to calculate the *t*-test statistic and mean ± SD. [43].

## Supplementary Material

Web_Material_uhaf138

## Data Availability

Raw reads for RNA-Seq were downloaded from NCBI Sequence Read Archive (SRA) database (https://www.ncbi.nlm.nih.gov/sra) under accession number PRJNA803327 (https://www.ebi.ac.uk/ena/browser/view/PRJNA803327).
